# Socio-demographic, psychosocial and home-environmental attributes associated with adults' domestic screen time

**DOI:** 10.1186/1471-2458-11-668

**Published:** 2011-08-25

**Authors:** Delfien Van Dyck, Greet Cardon, Benedicte Deforche, Neville Owen, Katrien De Cocker, Katrien Wijndaele, Ilse De Bourdeaudhuij

**Affiliations:** 1 Research Foundation Flanders (FWO), 1000 Brussels, Belgium; 2 Department of Movement and Sports Sciences, Ghent University, 9000 Ghent, Belgium; 3 Department of Human Biometrics and Biomechanics, Vrije Universiteit Brussels, 1000 Brussels, Belgium; 4 Baker IDI Heart and Diabetes Institute, 8008 Melbourne, Australia; 5 The University of Queensland, School of Population Health, QLD 4006 Brisbane, Australia; 6 MRC Epidemiology Unit, Institute of Metabolic Science, CB2 0QQ Cambridge, UK

**Keywords:** TV viewing time, leisure-time Internet use, Belgium, sedentary behavior, ecological model

## Abstract

**Background:**

Sedentary behaviors (involving prolonged sitting time) are associated with deleterious health consequences, independent of (lack of) physical activity. To inform interventions, correlates of prevalent sedentary behaviors need to be identified. We examined associations of socio-demographic, home-environmental and psychosocial factors with adults' TV viewing time and leisure-time Internet use; and whether psychosocial and environmental correlates differed according to gender, age and educational attainment.

**Methods:**

This cross-sectional study was conducted in Ghent, Belgium, between March and May 2010. Respondents to a mail-out survey (n = 419; 20-65 years; mean age 48.5 [12.1] years; 47.3% men) completed a questionnaire on sedentary behaviors and their potential socio-demographic, psychosocial and home environmental correlates. Statistical analyses were performed using multiple linear regression models.

**Results:**

The independent variables explained 31% of the variance in TV viewing time and 38% of the variance in leisure-time Internet use. Higher education, greater perceived pros of and confidence about reducing TV time were negatively associated with TV viewing time; older age, higher body mass index, larger TV set size and greater perceived cons of reducing TV time showed positive associations. Perceived pros of and confidence about reducing Internet use were negatively associated with leisure-time Internet use; higher education, number of computers in the home, positive family social norms about Internet use and perceived cons of reducing Internet use showed positive associations. None of the socio-demographic factors moderated these associations.

**Conclusions:**

Educational level, age, self-efficacy and pros/cons were the most important correlates identified in this study. If further cross-sectional and longitudinal research can confirm these findings, tailored interventions focusing on both psychosocial and environmental factors in specific population subgroups might be most effective to reduce domestic screen time.

## Background

Over the past years, much research has focused on the health benefits and determinants of regular physical activity (PA) in adults [1]. To gain insight into these determinants, ecological models of health behavior are frequently used as a theoretical framework [2]. These models identify factors at multiple levels (intrapersonal, socio-cultural, physical environmental and policy) and emphasize the importance of developing combined interventions to achieve behavior change. For PA, the most important determinants are becoming clear [3], which is helpful to develop effective interventions to increase PA and to prevent chronic diseases.

Recently, sedentary behaviors (involving prolonged sitting time; SB) also have been found to be associated with elevated risk of obesity, cardio-metabolic risk profile, diabetes, cardiovascular disease, specific cancers and overall and cardiovascular mortality [4-7]. These deleterious associations exist independent of the PA level and have been identified in cross-sectional and longitudinal studies addressing specific behaviors like television (TV) viewing, other screen-based entertainment and time spent sitting in cars, as well as in studies examining overall sitting time [4-11]. Because most adults spend more than 50% of their waking hours sedentary [12], interventions should not only aim at increasing PA, but also at decreasing SB in order to obtain optimal health effects.

Domestic screen time (TV viewing and leisure-time Internet use at home) has been related to negative health outcomes [7-9] and is one of the most prominent non-work related SBs of adults in developed countries [13]. Moreover, TV viewing and leisure-time computer use have been associated with higher levels of other leisure-time sedentary behavior [14,15]. Approximately 20% of the Belgian adults can be categorized as 'heavy TV watchers' (> three hours/day of TV viewing) [16] and 77.8% of the Belgian adult population has Internet access at home (http://www.internetworldstats.com 2010, Miniwatts Marketing Group; accessed June 13, 2011). Furthermore, changing these types of leisure-time SB might be more susceptible to individuals' own choice compared to for example occupational sitting. Therefore, TV viewing and leisure-time Internet use are target behaviors of interest when developing SB-reducing interventions. Nonetheless, before interventions can be developed, the crucial determinants of these behaviors need to be understood.

When investigating determinants and correlates of SB, ecological models of health behaviors can be used as a theoretical basis. However, since socio-demographic, psychosocial and physical environmental correlates of PA are poorly associated with SB, the PA-related ecological models should be adjusted, using SB-specific variables [17-19]. To our knowledge, very few studies have investigated possible specific correlates of TV viewing time and leisure-time Internet use in adults, so this type of research is still in an exploratory phase and the most important correlates remain to be clarified. Moreover, no studies have yet explored individual, psychosocial and environmental factors concurrently for associations with adults' SB.

Consequently the main aim of this study was to examine the associations of socio-demographic and sedentary-specific home-environmental and psychosocial factors with TV viewing time and leisure-time Internet use in Belgian adults. Moreover, since behavioral correlates can differ between men and women, younger and older adults and those of higher and lower educational attainment [20-22], an additional aim was to examine gender, age and educational level as potential moderators of these associations.

## Methods

### Participants and procedures

The present study was conducted in Ghent (237,000 inhabitants, 165.18 km^2^, 1468 inhabitants/km^2^), Belgium. Data were collected between March and May 2010. In total, 419 adults (20-65 years) participated in the study. To recruit participants, a questionnaire on SB and possible correlates and a letter explaining the purpose of the study were mailed to 1200 randomly selected (based on publicly available population data) urban and suburban adults. A prepaid and preaddressed envelope was provided to return the questionnaire. Fifty-three questionnaires (4.4%) were returned because people no longer lived at the address. After six weeks, 347 completed questionnaires were received and a second letter was sent to non-responders to ask for participation. After another six weeks, a total of 419 completed questionnaires (response rate 36.5%) was sent back. Informed consent was obtained from all participants and the study protocol was approved by the ethics committee of Ghent University Hospital.

### Measures

#### Self-reported domestic screen time

Domestic screen time included self-reported TV viewing time (min/day) and leisure-time internet use at home (min/day). Leisure-time internet use included visiting websites, emailing, and Facebook, Twitter, Netlog at home during leisure-time. These behaviors were determined using an adjusted version of the leisure-time SB questionnaire developed by Salmon and colleagues [23]. The original English-language questionnaire was translated into Dutch and instead of assessing the amount of SB during the last seven days, a 'usual week' version was used in this study. Assessing 'usual' behavior offers a more stable measure compared to assessing behaviors undertaken during the last seven days [24]. The English-language version of the questionnaire has fair to excellent reliability (intraclass-range from 0.56 to 0.82). Concurrent validity, assessed against a three-day behavioral log was fair-to-moderate with rho's ranging from 0.20 to 0.60 [23]. Duration (hours and minutes) of TV viewing and leisure-time Internet use on a regular weekday and weekend day was assessed. Daily means were calculated by the following formula: (weekday min/day*5 + weekend day min/day*2)/7.

#### Socio-demographic variables

These included self-reported gender, age, educational attainment (primary, secondary, tertiary education), employment status (employed, not employed/retired), and body mass index (BMI; calculated using self-reported height and weight). For the analyses, education was dichotomized into having college/university degree (yes/no).

#### Home-environmental variables

To assess the number of TVs and computers in the home, questions were derived from a previously validated home electronic equipment scale [25]. All participants recorded the number of TVs and computers (with Internet connection) available for use in their home. In addition, participants were asked about the size of their largest TV set. To obtain this information, five pictures of different TVs (screen diagonals ranging from 39 cm to 139 cm) were shown and participants were asked to indicate which TV resembled their own TV the most.

#### Psychosocial variables

All questions on psychosocial variables, except for social norm from family and friends, were derived from a validated questionnaire developed by Norman and colleagues [26] in the context of the PACE (Physician-based Assessment and Counseling for Exercise) study. This questionnaire has been mainly used in adolescent research, but since no specific psychosocial questionnaires have been developed for use in adults yet, it was used as a basis for the present adult study. Pros and cons of reducing screen time, self-efficacy about reducing screen time, and social norm from family and friends were included. The questions to assess social norm from family and friends were based on previously validated questions to measure social norms towards physical activity [27,28]. The wording of the original physical activity-related questions was changed to reflect social norm towards the targeted SBs. Table 1 shows the contents of the different questions. Computation of the relevant scales was based on the scoring protocol used in the PACE study [26]. Factor analyses confirmed the applicability of the original scoring protocol [26] to the present data. Internal consistency of the scales was moderate-to-good, with Cronbach Alpha values ranging from 0.50 to 0.78 (Table 1). All psychosocial correlates were rated on a five-point (one to five) Likert scale. For pros and cons of reducing screen time and social norm from family and friends, the response options ranged from 'strongly disagree' to 'strongly agree', while for self-efficacy about reducing screen time, the descriptors for the options ranged from 'I think this is very difficult' to 'I do not think this is difficult at all'.

**Table 1:** Sample characteristics and internal consistency of psychosocial factors

Variable	Total sample (n = 419)	Internal consistency Cronbach alpha
**Socio-demographic characteristics**		
Gender (%)		
Men	47.3	
Women	52.7	
Age (mean [SD])	48.50 (12.10)	
Educational level (%)		
No college/university degree	43.3	
College/university degree	56.7	
Employment status (%)		
Unemployed/retired	29.4	
Employed	70.6	
Occupation (%)		
Blue-collar	19.7	
White-collar	80.3	
Body Mass Index (mean [SD])	24.61 (3.90)	

**Home-environmental factors (mean [SD])**		
Number of TVs	1.65 (0.89)	
Size of largest TV set^a^	2.75 (1.04)	
Number of computers	1.75 (1.15)	

**Psychosocial factors (mean [SD])**		
Pros reducing TV viewing (sum of different items)^b^	2.59 (0.68)	0.50
*I think watching TV is boring*	2.51 (0.96)	
*Watching TV takes time away from doing other more important things*	2.80 (1.22)	
*I would feel lazy and sluggish if I watched TV for many hours*	3.52 (1.20)	
*Watching TV sometimes hurts my eyes and gives me a headache*	1.53 (0.85)	
Cons reducing TV viewing (sum of different items)^b^	2.54 (0.85)	0.77
*I enjoy watching TV for many hours at a time*	2.64 (1.13)	
*Watching TV is my way to escape from the world*	2.18 (1.09)	
*Watching TV is one of my favorite types of entertainment*	2.18 (1.14)	
*I find sitting and watching TV very relaxing*	3.17 (1.05)	
Family social norm TV viewing^b^		
*My family members think I spend too much time watching TV*	1.62 (0.90)	
Friends social norm TV viewing^b^		
*My friends think I spend too much time watching TV*	1.46 (0.72)	
Self-efficacy reducing TV viewing (sum of different items)^c^	3.76 (0.84)	0.73
*Turn off the TV even when there is a program on I enjoy*	3.25 (1.15)	
*Limit my TV time to one hour a day*	3.59 (1.27)	
*Leave the room where the TV is on even if others are watching TV*	4.20 (0.94)	
*Plan ahead of time what TV shows I will watch during the week*	4.01 (1.14)	
Pros reducing Internet use (sum of different items)^b^	2.51 (0.80)	0.50
*I think using the Internet is boring*	2.59 (1.18)	
*Using the Internet takes time away from doing other more important things*	2.40 (1.32)	
*I would feel lazy and sluggish if I used the Internet for many hours*	3.39 (1.37)	
*Using the Internet sometimes hurts my eyes and gives me a headache*	1.58 (0.95)	
Cons reducing Internet use (sum of different items)^b^	1.79 (0.74)	0.77
*I enjoy using the Internet for many hours at a time*	1.85 (0.99)	
*The Internet is my way to escape from the world*	1.65 (0.94)	
*Using the Internet is one of my favorite forms of entertainment*	1.66 (0.93)	
*I find sitting and using Internet very relaxing*	2.13 (1.12)	
Family social norm Internet use^b^		
*My family members think that I spend too much time using the Internet*	1.45 (0.78)	
Friends social norm Internet use^b^		
*My friends think that I spend too much time using the Internet*	1.31 (0.61)	
Self-efficacy reducing Internet use (sum of different items)^c^	4.14 (0.91)	0.78
*Turn off the computer even when I am doing something funny*	3.93 (1.10)	
*Limit my leisure-time Internet use to one hour a day*	4.38 (0.95)	
*Plan ahead of time how much time I will spend on leisure-time Internet use daily*	4.11 (1.18)	

**Sedentary behaviors (mean [SD])**		
TV viewing time (min/day)	128.40 (76.74)	
Leisure-time Internet use (min/day)	43.57 (46.57)	

^a ^Size of the largest TV was positively scored on a five-point scale (1-5), ranging from 39 cm to 139 cm

^b ^pros, cons, family social norm and friends social norm were positively scores on a five-point scale (1-5), ranging from 'strongly disagree' to 'strongly agree'

^c ^self-efficacy was positively scored on a five-point scale (1-5), ranging from 'I think this is very difficult' to 'I do not think this is difficult at all'

SD = standard deviation

### Data Analysis

All analyses were run using SPSS 16.0 for Windows. Multiple linear regression analyses were executed to investigate associations of socio-demographic, psychosocial and home-environmental factors with the outcome measures. Dependent variables were min/week of TV viewing time and min/week of leisure-time Internet use. Because both dependent variables were positively skewed, logarithmic transformations were applied to improve normality [29]. Independent variables included gender, age, education, employment status and BMI (socio-demographic variables), number of TVs/computers and size of largest TV set (home-environmental variables) and the following psychosocial variables: pros and cons of reducing TV viewing/Internet use (sum scores), self-efficacy about reducing TV viewing/Internet use (sum scores) and social norm from family and friends. To investigate whether the associations of the psychosocial and home-environmental factors with TV viewing time/leisure-time Internet use differed by gender, age and education, moderated multiple regression analyses were executed. To test for these potential moderating effects, the cross-product terms of gender/age/education with the different psychosocial and home-environmental factors were entered in a hierarchical multiple regression (block 2) after the main effects of the socio-demographic, home-environmental and psychosocial factors (block 1). To check the moderating effect of each factor, three regression models were constructed, one model for each potential moderator (gender/age/education) and its cross-products with the psychosocial and home-environmental factors. To avoid high correlations between the main effects and the interaction terms and reduce the effects of multicollinearity, centered variables (raw minus mean data) were used [30]. For all analyses, statistical significance was set at 0.05.

## Results

### Sample characteristics

Socio-demographic characteristics and mean scores for the home-environmental factors, psychosocial factors (sum scores and item level) and SB are shown in Table 1. Bivariate correlations of the home-environmental and psychosocial items with TV viewing time and leisure-time Internet use are presented in Table 2. The total sample consisted of 198 men (47.3%) and 221 women (52.7%). Mean age was 48.50 (12.10 years), mean BMI was 24.61 (3.90) kg/m^2^. Of all participants, 56.7% had a college/university degree, 70.6% was employed and 80.3% reported having a white-collar job. Compared with Belgian census data [31], the sample was more likely to be highly-educated and employed. Mean TV viewing time was 128.40 (76.74) min/day and the participants used Internet during leisure-time for on average 43.57 (46.57) min/day. Table 3 gives information on the amount of TV viewing time and Internet use according to gender, age and education. No significant correlation was found between TV viewing time and leisure-time Internet use (r = 0.029, p = 0.56).

**Table 2:** Bivariate correlations of home-environmental and psychosocial factors with sedentary behaviors

Variable	r _TV viewing_	r _Internet use_
**Home-environmental factors (mean [SD])**		
Number of TVs	0.15***	
Size of largest TV set^a^	0.13**	
Number of computers		0.18***
**Psychosocial factors (mean [SD])**		
Pros reducing TV viewing (sum of different items)^b^	-0.31***	
*I think watching TV is boring*	-0.29***	
*Watching TV takes time away from doing other more important things*	-0.13**	
*I would feel lazy and sluggish if I watched TV for many hours*	-0.35***	
*Watching TV sometimes hurts my eyes and gives me a headache*	0.02	
Cons reducing TV viewing (sum of different items)^b^	0.47***	
*I enjoy watching TV for many hours at a time*	0.54***	
*Watching TV is my way to escape from the world*	0.22***	
*Watching TV is one of my favorite types of entertainment*	0.36***	
*I find sitting and watching TV very relaxing*	0.30***	
Family social norm TV viewing^b^		
*My family members think I spend too much time watching TV*	0.34***	
Friends social norm TV viewing^b^		
*My friends think I spend too much time watching TV*	0.35***	
Self-efficacy reducing TV viewing (sum of different items)^c^	-0.49***	
*Turn off the TV even when there is a program on I enjoy*	-0.24**	
*Limit my TV time to one hour a day*	-0.65***	
*Leave the room where the TV is on even if others are watching TV*	-0.28**	
*Plan ahead of time what TV shows I will watch during the week*	-0.25**	
Pros reducing Internet use (sum of different items)^b^		-0.16**
*I think using the Internet is boring*		-0.39***
*Using the Internet takes time away from doing other more important things*		0.09
*I would feel lazy and sluggish if I used the Internet for many hours*		-0.19***
*Using the Internet sometimes hurts my eyes and gives me a headache*		-0.13*
Cons reducing Internet use (sum of different items)^b^		-0.31***
*I enjoy using the Internet for many hours at a time*		0.51***
*The Internet is my way to escape from the world*		0.30***
*Using the Internet is one of my favorite forms of entertainment*		0.49***
*I find sitting and using Internet very relaxing*		0.43***
Family social norm Internet use^b^		
*My family members think that I spend too much time using the Internet*		0.40***
Friends social norm Internet use^b^		
*My friends think that I spend too much time using the Internet*		0.26***
Self-efficacy reducing Internet use (sum of different items)^c^		-0.47***
*Turn off the computer even when I am doing something funny*		-0.30***
*Limit my leisure-time Internet use to one hour a day*		-0.54***
*Plan ahead of time how much time I will spend on leisure-time Internet use daily*		-0.51***

^a ^Size of the largest TV was positively scored on a five-point scale (1-5), ranging from 39 cm to 139 cm

^b ^pros, cons, family social norm and friends social norm were positively scores on a five-point scale (1-5), ranging from 'strongly disagree' to 'strongly agree'

^c ^self-efficacy was positively scored on a five-point scale (1-5), ranging from 'I think this is very difficult' to 'I do not think this is difficult at all'

r = Pearson correlation coefficient

*p < 0.05; **p < 0.01; ***p < 0.001

**Table 3:** Sedentary behaviors of the sample by gender, age and educational level

Socio-demographic	min/day TV viewing time		min/day leisure-time Internet use	
	
Factors	Mean (SD)	Median (IQR)	Mean (SD)	Median (IQR)
**Gender**				
Men (n = 198)	128.83 (78.88)	120.00 (120.00)	53.59 (53.17)	38.57 (40.71)
Women (n = 221)	128.02 (74.96)	120.00 (102.86)	34.67 (37.74)	25.71 (36.43)
**Educational level**				
No college/univ (n = 181)	165.95 (80.35)	180.00 (94.29)	44.31 (54.95)	30.00 (57.36)
College/univ (n = 238)	100.02 (59.96)	94.29 (81.43)	43.13 (39.20)	30.00 (45.00)
**Age**				
20 - 45 years (n = 169)	112.65 (72.57)	120.00 (77.14)	46.46 (46.10)	30.00 (40.71)
46 - 65 years (n = 250)	137.59 (41.94)	137.14 (102.86)	41.94 (46.91)	30.00 (49.82)

IQR = inter quartile range

SD = standard deviation

*Note: *medians and inter quartile ranges were reported because the data were strongly skewed

### Associations of socio-demographic, home-environmental and psychosocial variables with TV viewing time (Table 4)

The regression analyses showed that 39% of the variance in TV viewing time was explained by the independent variables. Regarding the socio-demographic factors, age (p < 0.001) and BMI (p = 0.030) were positively associated with TV viewing time, while educational level had a negative association (p = 0.001). Size of the largest TV set was the only home-environmental factor that was positively associated with TV viewing time (p = 0.012). For the psychosocial variables, perceiving more cons was associated with more TV viewing time (p = 0.014) while more pros (p < 0.001) and higher self-efficacy about reducing TV viewing time were related to less TV viewing time (p < 0.001). Results of the moderated regression analyses showed that none of the socio-demographic factors (gender, age, education) significantly moderated the associations between the psychosocial and home-environmental factors and TV viewing time (all p > 0.05).

**Table 4:** Multiple linear regressions on contribution of multidimensional correlates to TV viewing and leisure-time Internet use

Dependent variable	Adj R^2^	Significant correlates	Standardized β
TV viewing time	0.39	**Sociodemographic factors**	
		Age	0.189***
		Educational level	-0.148**
		Body Mass Index	0.096*
		**Home-environmental factors**	
		Size of largest TV set	0.107*
		**Psychosocial factors**	
		Pros reducing TV viewing	-0.177***
		Cons reducing TV viewing	0.155*
		Self-efficacy reducing TV viewing	-0.241***

Leisure-time Internet use	0.34	**Sociodemographic factors**	
		Educational level	0.112*
		Employment status	-0.154**
		**Home-environmental factors**	
		Number of computers	0.102*
		**Psychosocial factors**	
		Family social norm	0.161*
		Pros reducing Internet use	-0.116**
		Cons reducing Internet use	0.187**
		Self-efficacy reducing Internet use	-0.285***

Adj = adjusted

*p < 0.05; **p < 0.01; ***p < 0.001

### Associations of socio-demographic, home-environmental and psychosocial variables with leisure-time Internet use (Table 4)

In total, 34% of the variance in leisure-time Internet use was explained by the independent variables. Educational level was positively associated with leisure-time Internet use (p = 0.015), while employment status was negatively related to Internet use (p = 0.001). The number of computers was the only home-environmental factor that was positively associated with leisure-time Internet use (p = 0.020). Concerning the psychosocial factors, perception of higher social norm from family towards Internet use (p = 0.011) and more cons (p = 0.002) were related to more leisure-time Internet use. Moreover, more pros (p = 0.009) and higher self-efficacy about reducing leisure-time Internet use were associated with less Internet use (p < 0.001). Results of the moderated regression analyses showed that none of the socio-demographic factors (gender, age, education) significantly moderated the associations between the psychosocial and home-environmental factors and leisure-time Internet use (all p > 0.05).

## Discussion

This was the first study to investigate the concurrent contribution of socio-demographic, home-environmental and psychosocial factors to explain variance in domestic screen time in adults. Generally, the included factors explained a large amount (34% and 39%) of the variance in TV viewing and leisure-time Internet use. By conducting analyses that examined sociodemographic attributes as both predictors of the main effects, and as moderators of the relationships of home environmental and psychosocial factors with domestic screen time, we were able to examine specifically whether these associations differed by gender, age and educational attainment. The absence of interaction effects suggested that similar psychosocial and home-environmental factors can be targeted in different subgroups (men and women, younger and middle-aged adults, lower- and more highly-educated adults).

Detailed interpretation of the correlates showed that educational level and age were the strongest socio-demographic correlates of TV viewing time. Middle-aged and lower-educated adults watched more TV than younger and higher-educated adults. These findings are consistent with other adult studies [22,32-34] and consequently, one could plead for special attention to lower-educated and older adults when developing interventions to reduce TV viewing. A possible explanation for the findings could be that lower-educated adults, who spend most of their working day in manual tasks compensate by watching more TV in leisure-time. Previous study results have shown that occupational PA moderates the relationship between socio-economic status and leisure-time PA. Adults engaging in more PA during work were less active during leisure-time [35-37]. Consequently, they might have higher levels of leisure-time sitting, including TV viewing. Middle-aged adults might have more time to watch TV because they are less occupied (with childcare, work, etc) than are younger adults. Moreover, PA levels decrease with increasing age [1], so middle-aged adults might replace PA partly with more TV viewing time. Concerning leisure-time Internet use, lower-educated adults spent less time using Internet, which is opposite to the results of an Australian study [14]. Financial aspects may play a role: less-educated adults may have less financial resources and possibly have priorities other than buying a computer with Internet connection for leisure-time use. Our findings support this assumption: the prevalence of having no computer with Internet access at home was higher in less-educated adults compared to higher-educated adults (13.3% and 4.6% respectively, χ^2 ^= 9.9, p = .002). Unemployed and retired adults spent more time using Internet in the present study; this could potentially be explained by the higher discretionary time they have available [38]. BMI was less consistently associated with the outcome measures. Only for TV viewing, a positive association was found. This was unexpected, as other studies in adults consistently showed strong positive associations of BMI with TV viewing time and leisure-time Internet use [14,22,33]. The non-significant association between BMI and internet use might be partly explained by the lower prevalence of this behavior compared with TV viewing. Moreover, several other factors besides screen time, like snacking, total caloric intake and PA might be more strongly related to BMI [39].

Regarding the home-environmental factors, positive associations were found between the size of the largest TV set and TV viewing time and between the number of computers and leisure-time Internet use, although the beta-values were small. However, no association was found between the number of TVs and TV viewing time. Roemmich and colleagues [40] found the number of TVs in the home to be positively associated with TV viewing time in adolescent girls. It might be that in adults, having TVs versus having no TVs shows a stronger association with SB than just the number of TVs. Since only a very small percentage of the present sample (2.9%) had no TV in the home, this could possibly explain the non-significant results. Moreover, the location of the TV (bedroom vs other location) might also be important in the association with TV viewing time [41]. Overall, no strong evidence was found for the importance of home-environmental factors to explain variance in domestic screen time in this study.

Psychosocial factors were strongly associated with domestic screen time. Self-efficacy about reducing screen time and perceiving pros and cons to reduce screen time were the most consistent correlates. No other adult studies examining these factors were identified, but in adolescents, self-efficacy and decisional balance (pros - cons) have been found to be important correlates of total sedentary time [26,42]. When examining the psychosocial constructs on item level, several consistent correlations with the outcome measures emerged. For self-efficacy, "confidence in being able to limit TV viewing time/Internet use to one hour a day" was strongly correlated to the outcome measures, while "thinking that TV viewing/Internet use is boring" and "feeling lazy and sluggish if watching TV/surfing on Internet for many hours" were the most strongly related pros. Concerning the cons, all items had strong correlations with TV time and Internet use. This specific information can be helpful to decide on which specific constructs one could focus when developing tailored SB-reducing interventions. Family social norm was associated with leisure-time Internet use, but not in the expected direction. A possible explanation for this unexpected result could be the phrasing of the questions on social norms. The questions might assess behavior rather than attitudes towards behavior. Consequently, the amount of domestic screen time might determine the responses to these questions, biasing the results. Reformulation of these questions should be considered in further studies. Furthermore, the questions to assess social norm consisted of only one item for family and one item for friends and were derived from questions to measure social norm towards physical activity; thus, our findings might have differed if this factor would have been assessed using multiple items.

When comparing the present findings to what has been reported in studies examining correlates of PA in adults, similar patterns can be identified. Self-efficacy is one of the most consistent correlates of PA, together with perceived benefits and barriers and social support [1]. Moreover, psychosocial factors (because they can be assessed in ways that closely match the behaviors of concern) usually explain a larger proportion of the variance in PA than do environmental attributes [43]. These similarities suggest that the use of behavior-specific correlates may lead to some findings that are comparable across different behavioral domains. This is interesting for future interventions, since focusing on self-efficacy and benefits/barriers towards health behaviors (including both PA and SB) might lead to positive effects on different outcomes.

The first study limitation was the cross-sectional study design, inducing that no inferences on causality can be made. Second, both outcome measures and potential correlates were self-reported, so the measures may suffer from social desirability and underestimation. Third, since the study sample was somewhat more likely to be highly-educated and employed compared to the Belgian population, generalizability of the results may be limited. Fourth, some possibly important correlates (e.g. number of TVs in the bedroom, enjoyment of SB, social support towards decreasing SB) were not included in the questionnaire or were only assessed with a limited number of questions (as was the case for how we assessed social norm from family and friends). These factors should be investigated more thoroughly in the future. Moreover, the validity of questions to assess social norm from family and friends towards domestic screen time needs to be examined. Strengths of this study included the use of validated psychosocial questionnaires and the inclusion of correlates of multiple domains.

In general, these findings are promising, with high proportions of variance in domestic screen time explained by mainly socio-demographic and psychosocial correlates. Future research should keep this focus on different SB (not only TV viewing and leisure-time Internet use, but also automobile-sitting and job-related sitting) and on multiple levels of determinants, because a wide range of correlates can be important for different types of SB. Moreover, within the ecological perspective [2], other correlates like modeling, enjoyment of SB and neighborhood factors could be included in future correlation models to increase the explained variance of SB [44]. According to the behavioral epidemiology framework of Owen and colleagues [18] research should try to improve the measurement of SB (increased focus on objective measurement tools like accelerometers) and to strengthen the findings of behavioral determinants studies and intervention trials to reduce these behaviors.

## Conclusions

In summary, the present findings show that educational level, age, self-efficacy about reducing screen time and pros and cons for reducing screen time were the most important correlates of TV viewing time and leisure-time Internet use in a sample of Belgian adults. Based on these findings, tailored interventions focusing mainly on psychosocial factors in specific population subgroups might be most effective to reduce domestic screen time. If SB-reducing interventions targeting the most-relevant determinants were to be combined with strategies to increase PA and to develop healthy eating habits in the future, health effects could be reached in large populations.

## Conflict of interest statement

The authors declare that they have no competing interests.

## Authors' contributions

All authors contributed to the design of different parts of the study. DVD was responsible for data collection, performed the statistical analyses and drafted the manuscript. IDB, GC and BD helped to prepare the data collection, participated in the interpretation of the data, helped to draft the manuscript and revised the manuscript for important intellectual content. NO, KDC and KW revised the draft for important intellectual content. All authors read and approved the final manuscript.

## Pre-publication history

The pre-publication history for this paper can be accessed here:

http://www.biomedcentral.com/1471-2458/11/668/prepub
